# TDO2 Promotes the EMT of Hepatocellular Carcinoma Through Kyn-AhR Pathway

**DOI:** 10.3389/fonc.2020.562823

**Published:** 2021-01-19

**Authors:** Lei Li, Tao Wang, Shanbao Li, Zhengqian Chen, Junyi Wu, Wanyue Cao, Qi Wo, Xuebin Qin, Junming Xu

**Affiliations:** ^1^Department of General Surgery, Shanghai General Hospital, Shanghai Jiao Tong University School of Medicine, Shanghai, China; ^2^Division of Pathology, Tulane National Primate Research Center, Health Sciences Campus, Covington, LA, United States

**Keywords:** hepatocellular carcinoma, metastasis, epithelial to mesenchymal transition (EMT), Tryptophan 2,3-dioxygenase (TDO2), aryl hydrocarbon receptor (AhR)

## Abstract

Tryptophan 2,3-dioxygenase (TDO2), an enzyme involved in tryptophan (Trp) metabolism has been linked with some malignant traits of various cancers. Kyn, the main product of Trp metabolism pathway catalyzed by TDO2 and indoleamine 2,3-dioxygenase (IDO) in tumor cells, was also demonstrated to activate aryl hydrocarbon receptor (AhR), which may regulate cancer growth and invasion in some malignancies. However, whether TDO2 participates in the metastasis and invasion of HCC has not been explored before. The underlying mechanism played by TDO2 in this process still requires further investigation. Here, we demonstrated that overexpression of TDO2 correlates with advanced stage or malignant traits in HCC patients. Knockdown or inhibition of TDO2 suppressed the migration and invasion of HCC cells *in vitro* and *in vivo*. Epithelial to mesenchymal transition (EMT) is an essential program happened in the initial phase of cancer metastasis. We found that in HCC cells, TDO2 promoted the EMT process evidenced by altered levels of biomarkers for EMT. Mechanically, TDO2 regulated the Kyn production in HCC cell *via* activated aryl hydrocarbon receptor (AhR). Together, these results indicate that TDO2 promotes the EMT of hepatocellular carcinoma through activating Kyn-AhR pathway, thereby participating in the metastasis and invasion of HCC.

## Introduction

Liver cancer, the fifth most common cancer, ranks the second leading cause of cancer-related death worldwide (1, 2). Hepatocellular carcinoma (HCC) is the major forms of primary liver cancer. Overall prognosis for HCC patients remains poor due to the highly metastatic and aggressive biological features of HCC, which leading to advanced clinical stages and high recurrence rate of HCC patients (3, 4). Although molecular mechanisms underlying HCC metastasis has drawn a great deal of attention, it still remains unclear and requires further investigation.

Tryptophan 2,3-dioxygenase (TDO2), encoded by gene Tdo2, is expressed normally at high levels in the liver. It acts as the first and rate-limiting step of tryptophan (Trp) metabolism along kynurenine (Kyn) pathway and maintains systemic tryptophan levels (5). Kyn, the main product of Trp metabolism pathway catalyzed by TDO2 and indoleamine 2,3-dioxygenase (IDO) in tumor cells, was demonstrated to activate aryl hydrocarbon receptor (AhR), suppressing antitumor immune responses and promoting tumor-cell survival and motility through AhR in an autocrine/paracrine fashion (6). AhR is identified as a ligand-activated transcription factor of the basic helix-loop-helix (bHLH) Per-Arnt-Sim (PAS) family and also plays an essential role in a wide range of physical and pathological condition (7). TDO2 is constitutively expressed in various cancer cells, such as hepatocarcinoma, bladder carcinoma, breast carcinoma, colorectal carcinoma, lung carcinoma, and glioblastoma, playing a role in immune surveillance and tumor biology (6, 8, 9). Recent studies revealed that TDO2 affects biological features directly in different cancers (6, 9, 10). TDO2 is highly expressed in HCC, however, whether TDO2 participates in the metastasis and invasion of HCC has not been explored before. Further, the underlying mechanism played by TDO2 in this process in HCC still requires further investigation.

Epithelial to mesenchymal transition (EMT), a process that epithelial cells lose their polarized organization and acquire migratory and invasive capabilities, is considered to contribute to cancer metastasis (11, 12). Therapeutics targeted to EMT pathway show a great potential for preventing tumor dissemination or sweeping off metastatic cancer cells in patients in advanced stage (13, 14). EMT biomarkers, such as Vimentin, N-Cadherin and MMP9 are overexpressed on HCC and participate in facilitating the metastasis of HCC (15–18). These suggest that induction EMT contributes to the acquisition of high metastatic trait of HCC. Many studies revealed that AhR activity contributed to the loss of cell contact-inhibition and altering extracellular matrix remodel (19). Considerable evidence has been piled up supporting the critical role of AhR activation in the induction of EMT (19–22). Previous study showed that AhR was overexpressed in HCC and associated with its tumorigenesis and invasion (23, 24). These results prompted us to hypothesis that TDO2 may contribute to tumorigenesis and metastasis and invasion of HCC *via* activation of AhR leading to increased EMT.

Here, we report our studies of the role of TDO2 in the metastasis and invasion of HCC, we searched TCGA database and mined data of the expression of TDO2 in different cancers. We found that the expression level varied in HCC and stomach adenocarcinoma according to different stages. The TDO2 expression was relatively high in HCC with vascular invasion and so was it in stomach adenocarcinoma with advanced stages in clinical samples. The effect of knockdown or inhibition of TDO2 on the EMT associated metastasis ability of HCC cell lines was investigated by *in vitro* and vivo experiments. Mechanistically, we demonstrated that TDO2 was responsible for the metabolism of Trp along Kyn pathway in HCC cells, and regulated the EMT process at least partly through Kyn-AhR pathway. Together, our results indicate that the overexpression of TDO2 promotes HCC metastasis capability through Kyn-AhR mediated induction of EMT.

## Materials and Methods

### Materials

Huh7 and LM3 HCC cell lines were transfected with two puro plasmid expressing sh-Tdo2 and scrambled control using transfection reagent (provided by Haro Life, Shanghai, China). Two shRNAs were designed to knockdown of TDO2, shown as following.

TDO2 inhibitor 680C91, Tryptophan and Kynurenine were purchased from Sigma-Aldrich, AhR inhibitor CH-223191 were purchased from Selleck Chemicals (Houston, TX, USA).

pLKO.1puro-shhTDO2-AF

CCGGGGAAAGAACTCCAGGTTTACTCGAGTAAACCTGGAGTTCTTTCCTTTTT

pLKO.1puro-shhTDO2-AR

AATTAAAAAGGAAAGAACTCCAGGTTTACTCGAGTAAACCTGGAGTTCTTTCC

pLKO.1puro-shhTDO2-BF

CCGGTCATAAGGATTCAGGCTAACTCGAGTTAGCCTGAATCCTTATGATTTTT

pLKO.1puro-shhTDO2-BR

AATTAAAAATCATAAGGATTCAGGCTAACTCGAGTTAGCCTGAATCCTTATGA

### Specimens and Cell Culture

All clinical specimens were obtained with informed consent of patients in Shanghai general hospital and confirmed by pathologists. Twenty-three cases of paired specimens, HCC tissue and the adjacent normal tissue, and 16 cases of gastric cancer tissue were collected and stored in liquid nitrogen. Another 28 cases of HCC specimens were collected and stored in formalin. Human liver cancer cell lines (Huh7, LM3, Hep3B, HepG2, 97H-GFP-LC3) and normal human liver cell (LO2) were obtained from Type Culture Collection of the Chinese Academy of Science (Shanghai, China). Cell lines except for HepG2 were cultured in DMEM medium and HepG2 in MEM medium, with 10% fetal bovine serum (FBS) (Gibco, Grand Island, NY, USA) and 1% penicillin-streptomycin under a humidified atmosphere containing 5% CO2 at 37°C.

### Migration and Invasion Assays

Scratch wound assays and Transwell chamber assays were applied to test the migration and invasion capabilities of HCC cells. For scratch wound healing assay, 5×10^5^ cells were firstly seeded per well in 6-well plates and cultured for 24 h, after which scratching with a 200 μl micropipette tip in the center of the well were performed. Then, the cells were cultured with serum-free medium and corresponding treatment. Images were captured at 0 and 48 h after scratch. The width of wound healing was measured, and migration rate was calculated.

Transwell chamber assays with and without Matrigel-coated were performed to show cell migration and invasiveness. Cells were seeded at 20,000 cells or 40,000 per well in DMEM medium in the upper chamber without or with Matrigel coated, respectively. Six hundred μl Medium containing 10% FBS was added to the bottom chamber. Forty-eight hours later, cells in the upper surface of transwell chamber were erased with swab and cells transferred to the lower surface of the chamber were fixed in 4% paraformaldehyde and then stained with aniline violet for visualization and photography.

Three independent experiments were carried out. Mean ± standard error of mean (SEM) was calculated for each of the experiments.

### Western Blot Analysis

Specimens and Cells were lysed in RIPA with 1% PMSF. Nuclear and cytoplasmic separation were guided according to the manual of Nucleo-cytoplasmic protein extraction kit (Thermo Scientific, USA). Cell protein extracts (50 μg) were denatured by boiling, separated on SDS-PAGE gels, and electrotransferred to polyvinylidene fluoride membranes (Millipore 0.45 um, USA). After the membranes were blocked with 5% skim milk dissolved in Tris-buffered saline containing 0.1% Tween20, they were incubated with primary antibodies overnight at 4°C, including anti-TDO2 (NOVUS, USA), anti-AhR (Abcam, UK), anti-Cyp1b1(Abcam, UK), anti-E Cadherin(CST, USA), anti-N Cadherin(CST, USA), anti-MMP9 (CST, USA), or anti-Vimentin antibodies(CST, USA). The antibodies above were applied at the concentration of 1:500. Horseradish peroxidase-conjugated anti-rabbit IgG or anti-mouse IgG was used as secondary antibodies, at the concentration of 1:2,000. Immunocomplexes were visualized with an ECL luminescence reagent (Absin, China). Glyceraldehyde 3-phosphate dehydrogenase (GAPDH, 1:1,000 dilution, Proteintech, USA) was used as an internal control. Densitometry quantification was performed using Image J.

### Quantitative RT-PCR

Total RNA was isolated using the traditional Trizol methods. cDNA was synthesized with ProFlex™ PCR system using PrimeScript™ RT Master Mix reagent kit (TaKaRa, Shiga, Japan). Quantitative reverse transcription PCR (qRT-PCR) was performed in Roche LightCycler 96 using SYBR Premix Ex Taq™ (TaKaRa, Shiga, Japan). All fold-change data were normalized to GAPDH. The 2-ΔΔCt method was used to calculate relative expression levels.

### Immunohistochemistry

The HCC tissue paraffin sections were subjected to de-paraffinization in xylene, rehydration through graded ethanol (100, 95, 85, 80, 75%) and distilled water, prior to boiling in 10 mM citrate buffer solution (pH 6.0) for 15 min for antigen retrieval. Three percent H_2_O_2_ was applied to incubate the tissue arrays for 10 min to quench endogenous peroxidase activity. After blocked with 1% bovine serum albumin for 20 min, tissue arrays were incubated with primary antibody, including TDO2 antibody, anti-N cadherin or anti-E cadherin, with 1:100 dilution overnight at 4°C, prior to incubation with biotinylated secondary antibody for 30 min at 37°C. Coloration lasted for 1 min in DAB (Invitrogen, USA). Images were recorded using Lax software under the unified parameters.

### Immunofluorescence Microscopy

Cells were fixed with 4% paraformaldehyde in PBS for 10 min at room temperature. Cells were permeabilized with 0.3% TritonX-100 in PBS for 20 min and then blocked with 1% BSA in PBS containing 0.3M glycine. Subsequently, the primary anti-AhR antibody (1:100 dilution) was added to the cells and incubated overnight at 4°C. The secondary FITC combined antibody (Solarbio, China) was diluted in 1:100 and added to cells for 30 min at room temperature. 40,6-diamidino-2-phenylindole (DAPI) was last added for 5 min to visualize the nuclear of cells. Immunostaining was observed at 400 magnification using the Leica TCS SP8 confocal microscope and images were captured using the Leica LAS-AF software (Leica Microsystems, Germany).

### Analysis of Tryptophan and Kynurenine

High performance liquid chromatography (HPLC) was used to analyze the concentration of Trp and Kyn. Cell culture supernatant was collected, centrifuged, and transferred to fresh tubes and frozen until subjected to analysis. Two hundred μL sample was precisely pipetted, and 200 μM perchloric acid was added for purification. The sample was mixed with vortex for 30 s and placed at room temperature for 5–10 min, followed by centrifugation for 5 min at 10,000 r/min. The supernatant was collected for test. VWD C18 column (250*4.6mm;5μl) was used as detector. Twenty μl sample was injected with the speed of 1.0 ml/min and measured at 225 nm wavelength. Fifteen mmol/L acetic acid: sodium acetate (containing 2.7% acetonitrile, PH = 3.6) was applied as mobile phase. The concentrations were calculated based on standard solutions.

### *In Vivo* Models

A total of 20 BALB/c nude mice were used for the orthotopic mouse model of HCC according to previous study (25). Mice were anesthetized by isoflurane, and 2×10^6^ cells in 25μl PBS containing 25% Matrigel were injected into the subcostal region of the left lobe. Mice were sacrificed 6 weeks later, and the livers were removed, imaged, and embedded in paraffin. Hematoxylin and eosin staining were performed to confirm tumor metastasis. All animal experiments were approved by the Institutional Animal Care and Use Committee of Shanghai General Hospital.

### Statistical Methods

Statistical analyses were carried out and graphics were generated using GraphPad Prism 7.00. Results are shown as representative images or as mean ± SEM of at least three independent experiments. Data according with Gaussian distribution were analyzed using the unpaired t-test. The basic information parameters of patients were analyzed using Fisher’s exact test. Data shown in graphical format represented as means (± SEM) or medians with interquartile range. *P* value <0.05 is considered statistically significant.

## Results

### Overexpression of TDO2 Was Associated With Advanced Stage or Malignant Traits in Patients With HCC and Stomach Carcinoma

**Figure 1:**
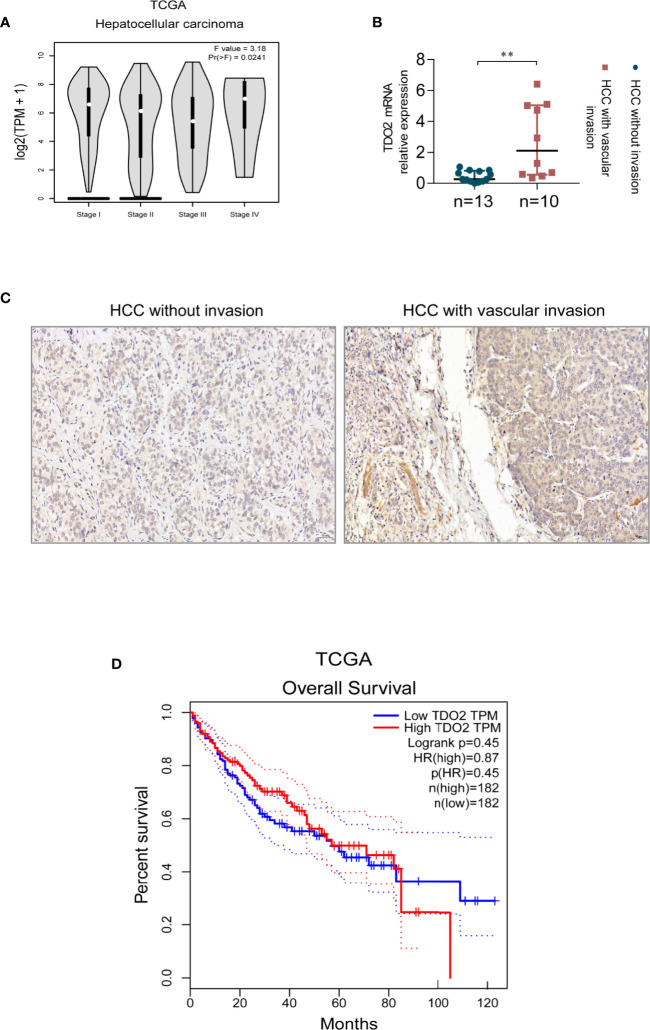
The expression level of TDO2 correlates with advanced stage or malignant traits of carcinoma. **(A)** TDO2 expression in HCC of different stages shown by data from TCGA. **(B, C)** TDO2 mRNA and protein level in HCC samples with and without vascular invasion measured by qRT-PCR and IHC, respectively. **(D)** The relationship between TDO2 expression and overall survival of HCC patients according to the data obtained from TCGA database. **P < 0.01. Scale bar = 50 μm. The graphs were derived from the website GEPIA (26).

To investigate whether the expression of TDO2 correlates with HCC progress and other digestive maliganancies, we utilized the public data available in TCGA (The Cancer Genome Atlas) database. We found that stomach adenocarcinoma and esophageal carcinoma in advanced stages had higher TDO2 expression than that in early stages (**Supplementary Figure 1A**). As for HCC, the public data also showed an upregulated expression of TDO2 in cancer of metastasis compared with that without metastasis (**Figure 1A**). We used qRT-PCR and IHC to analyze TDO2 mRNA and protein levels in 23 pairs of HCC samples and adjacent normal tissues. We demonstrated that HCC with vascular invasion had higher TDO2 expression at transcriptional and translational level than HCC without vascular invasion (**Figures 1B, C**). Besides, by Western Blot analysis of clinical gastric carcinoma samples (n = 8), we also found that the expression level of TDO2 was relatively higher in gastric carcinoma in stage III–IV than in stage I–II (**Supplementary Figure 1B**). And the analysis of co-relationship of TDO2 expression level and overall survival of HCC patients showed a shorter survival time in high TDO2 group than in low TDO2 group, while no statistical significance reached (**Figure 1D**). These results indicate that the upregulated expression of TDO2 is related to malignancy grade, which may contribute to the invasion and metastasis of HCC, and further influencing the prognosis of patients.

### Knockdown or Inhibition of TDO2 Decreased the Migration and Invasion Capabilities of HCC Cell Lines *In Vitro* and *Vivo*

**Figure 2:**
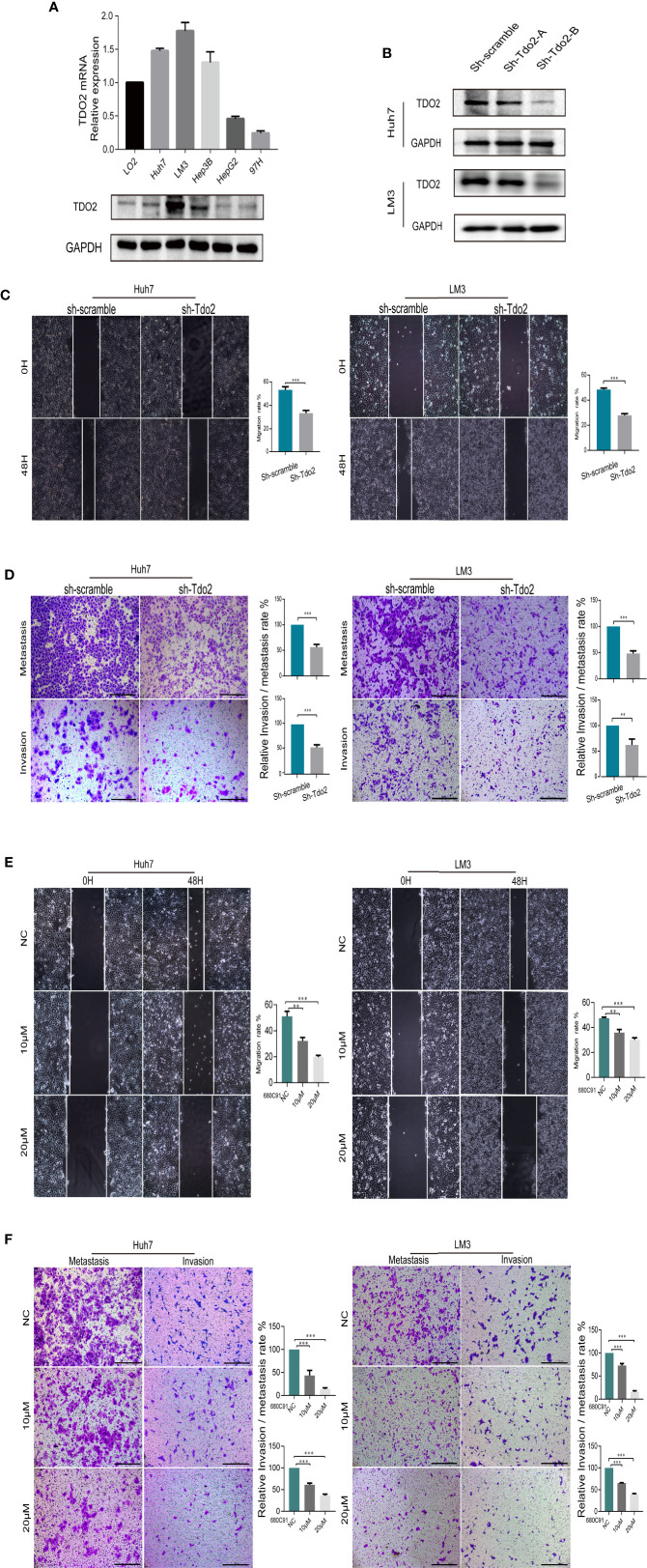
TDO2 enhanced the metastasis of HCC cells *in vitro* and vivo. **(A)** Relative expression of TDO2 in HCC cell lines compared to normal liver cell LO2, as shown by qRT-PCR and Western Blot. **(B)** Establishment of TDO2 knockdown cell lines in Huh7 and LM3 cells, confirmed by Western Blot. **(C)** Representative data from Scratch wound assays performed with sh-Tdo2 and sh-scramble groups in Huh7 and LM3 cells. The migration distance, that is the difference between the width of wound at 0 h and that at 48 h measured using Adobe illustrator software, was recorded, and the migration rate, namely the ratio of migration distance to the initial wound width, was calculated. **(D)** Representative data from Transwell migration and Matrigel invasion assays with indicated cells. **(E)** Representative data from Scratch wound assays performed with the Huh7 and LM3 cell lines treated with TDO2 inhibitor 680C91 at different concentration. **(F)** Representative data from Transwell migration and Matrigel invasion assays with Huh7 and LM3 cells treated with TDO2 inhibitor 680C91 at different concentration. All data were recorded as means ± SEM of three independent experiments. Scale bar = 200 μm. **P < 0.01; ***P < 0.001.

To examine the role of TDO2 in promoting the development of HCC, we used both knockdown and inhibitory approaches to suppress the TDO2 activity in 5 HCC cell lines. The level of TDO2 expression level was upregulated in LM3, Huh7 and Hep3B cell lines as compared with that in the immortalized normal human liver cell line LO2, at both protein and mRNA levels (**Figure 2A**). Then two shRNA sequences packed with effective lentivirus were designed and utilized to knockdown Tdo2 gene in Huh7 and LM3 cells, while only one of them showed knockdown effects (**Figure 2B**). Thus, cells transferred with sh-Tdo2-B were used for the following experiments as a knockdown group (or sh-Tdo2 group). Scratch wound assays and Transwell assay showed that sh-Tdo2 groups in both Huh7 and LM3 cells has significantly reduced capabilities of the migration and invasion than sh-scramble groups (P < 0.01, **Figures 2C, D**). In addition, to inhibit the TDO2 in these cells, we applied 680C91, a specific inhibitor used for suppressing TDO2 activity (5) to these cells at 10 or 20 μM concentrations. Consistently, the inhibition of TDO2 also suppressed the migration and invasion capabilities of Huh7 and LM3 (P < 0.01, **Figures 2E, F**).

**Figure 3:**
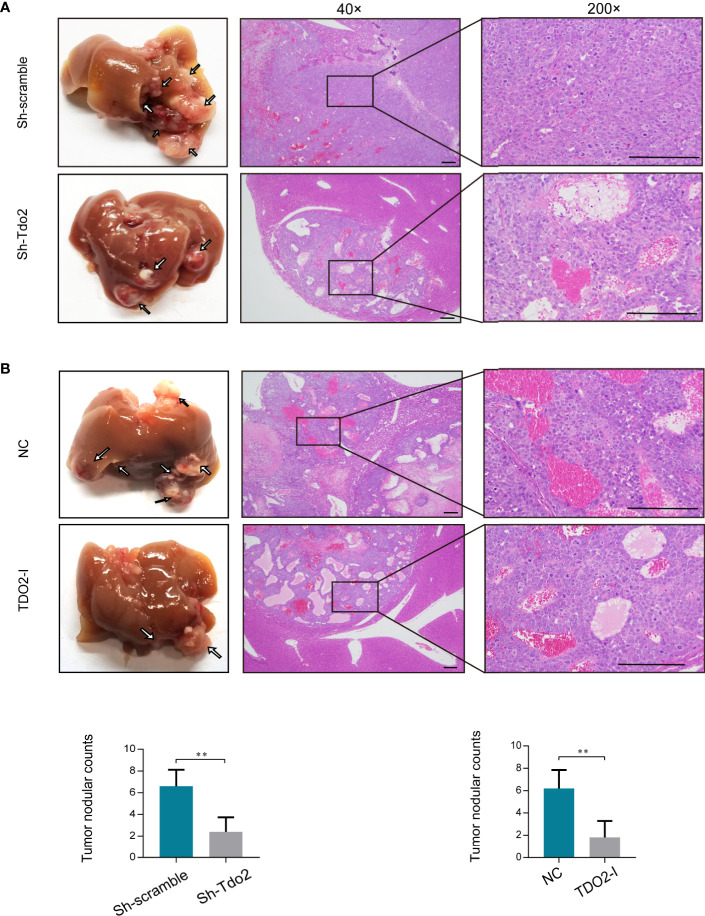
**(A)** Knockdown of TDO2 decreased intrahepatic tumor metastasis of Huh7 cells in mice. **(B)** Inhibition of TDO2 by inhibitor 680C91 decreased intrahepatic tumor metastasis of Huh7 cells in mice. Photo micrograph of HE-stained tissues that showed metastasized HCC cell masses (magnification×4 and ×200, Scale bar = 200 mm). **P < 0.01.

We further used HCC orthotopic model in nude mice to evaluate the effect of TDO2 on metastasis of HCC cells *in vivo*. Huh7 cell line was utilized for developing the *in vivo* model since it developed the satisfactory characteristics of tumorigenesis in this model. Both knockdown of TDO2 in Huh7sh-Tdo2 cells and inhibition of TDO2 in the Huh7 cell lines treated with 680C91 group developed less metastatic nodules than their respective control groups (**Figures 3A, B**). The above results indicate that TDO2 participates in the migration and invasion of HCC cells both *in vitro* and vivo.

### TDO2 Promoted the Epithelial-To-Mesenchymal Transition Process in Hepatocellular Carcinoma Cells

**Figure 4:**
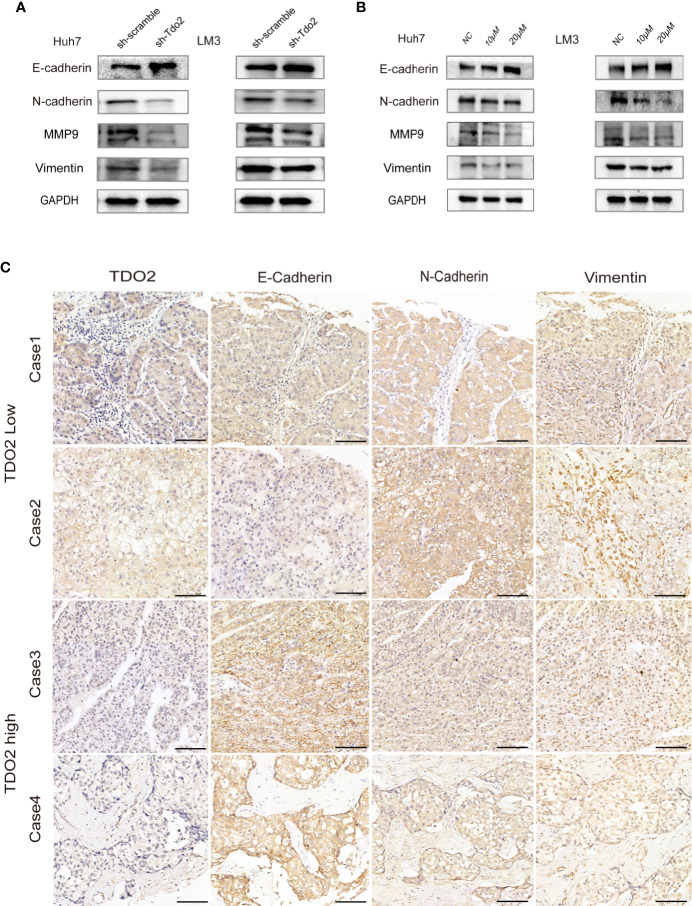
TDO2 could promote EMT process in HCC cells. **(A)** Relative expression levels of E-cadherin, N-cadherin, Vimentin, and MMP9 in sh-Tdo2 and sh-scramble groups in Huh7 and LM3 cells. **(B)** Relative expression levels of E-cadherin, N-cadherin, Vimentin, and MMP9 in Huh7 and LM3 cells treated with TDO2 inhibitor 680C91. **(C)** Representative pictures of IHC of E-cadherin, N-cadherin, and Vimentin comparing tissues with high TDO2 levels and those with low TDO2 levels. Scale bar = 100 μm.

We further investigated whether TDO2 overexpression promoted metastasis by modulating EMT of these HCC cell lines. EMT occurs during tumor progression to the metastatic phenotype. E-cadherin, a key marker of the epithelial phenotype, is a transmembrane protein responsible for cell-cell contact and adherence junction, the loss of which is considered as a key step for metastasis (27). N-cadherin and Vimentin, two proteins considered to be markers of a mesenchymal phenotype and crucial for cellular migration, are upregulated during EMT (15, 16). MMPs, such as MMP2 and MMP9, are upregulated markers during EMT that are capable of helping migratory cells to invade neighboring tissues and break through the basement membrane by cleaving cell-surface proteins and degrading components of extracellular matrix (28). Therefore, we utilized these EMT-associated markers (E and N-cadherin, MMP9 and vimentin) to assess EMT status of these HCC cells. We documented that in sh-Tdo2 and inhibitory groups of Huh7 and LM3 cells, an increased expression of E-cadherin, as well as decreased expressions of N-cadherin, MMP9, and Vimentin, was observed compared to negative control groups (**Figures 4A, B**). This result indicates that knockdown or inhibition of TDO2 impeded EMT process in HCC cells. IHC assay was performed to compare the expression levels of E-cadherin, N-cadherin, and Vimentin in HCC samples with different levels of TDO2. HCC tissue with low TDO2 expression level showed higher level of E-cadherin and lower level of N-cadherin and Vimentin, whereas samples with high TDO2 expression level showed relatively lower level of E-cadherin and higher level of N-cadherin and Vimentin (**Figure 4C**). These data suggested that TDO2 overexpression promoted EMT to facilitate metastasis in HCC.

### TDO2 Promoted Epithelial-To-Mesenchymal Transition Process *via* Kyn-AhR Pathway

**Figure 5:**
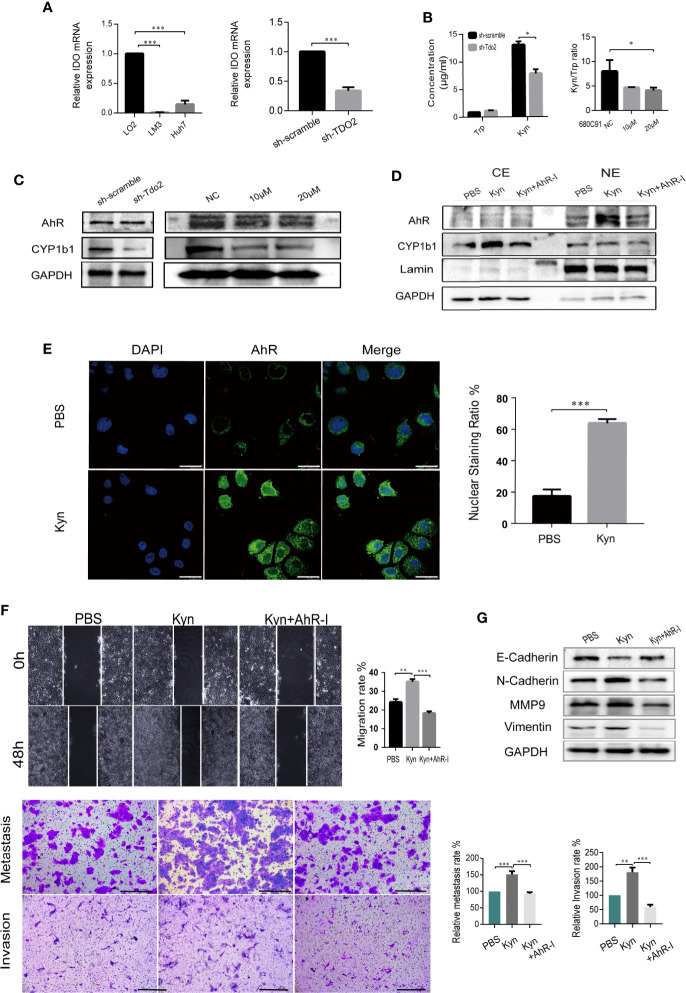
TDO2 promoted EMT process *via* Kyn-AhR pathway. **(A)** The relative expression of IDO in LM3 andHuh7 compared to normal liver cell LO2, and in sh-Tdo2 Huh7 cells compared to sh-Scramble Huh7 cells, verified by qRT-PCR. **(B)** The concentration of Trp and Kyn in the supernatant of sh-scramble and sh-TDO2 Huh7 cells and the ratio of Kyn/Trp in the supernatant of Huh7 cells treated with TDO2 inhibitor 680C91. The concentration of Trp and Kyn was measured by HPLC. **(C)** Relative expression levels of AhR and its target gene CYP1b1 in the indicated cells. **(D, E)** Translocation of AhR activated by exogenous Kyn observed by Western blot and laser confocal fluorescence microscopy, Scale bar = 25 μm. **(F)** Scramble cell assay and Transwell metastasis and invasion assay of sh-Tdo2 Huh7 cells treated with PBS, Kyn (50 μM) and Kyn (50 μM) combined with AhR inhibitor CH-223191 (10 μM), Scale bar = 200 μm. **(G)** Relative expression levels of E-cadherin, N-cadherin, Vimentin and MMP9 in the sh-Tdo2 Huh7 cells treated with Kyn and AhR inhibitor measured by Western Blot. *P < 0.05; **P < 0.01; ***P < 0.001.

We further explored the molecular mechanism underlying TDO2-promoted EMT process. TDO2 was the main enzyme catalyzing Tryptophan in HCC cell lines, as the expression of IDO was relatively low (**Figure 5A**). Therefore, we further investigate whether TDO2 promoted EMT process *via* Kyn-AhR pathway. We tested whether TDO2 knockdown or inhibition affected Kyn production in Huh7 cell line. TDO2 knockdown increased Trp accumulation and decreased Kyn level. Consistently, Kyn/Trp ratio, a marker routinely used for measuring the activity of Trp metabolic enzymes, TDO2 and IDO (29), was decreased while TDO2 inhibitor was applied to the cells (**Figure 5B**). TDO2 knockdown or inhibition abrogated the abundance of CYP1b1, which indicated weakened activity of AhR (**Figure 5C**). Treating Huh7 cells with 50 μM exogenous Kyn, AhR was observed to be activated and transferred to the nuclei by Western Blot (**Figure 5D**) and further verified by fluorescence confocal microscope (**Figure 5E**), demonstrating that Kyn mediated the activation of AhR in Huh7 cells.

Besides, public data showed a positive correlation between the level of TDO2 and that of AhR in malignant tumors, including colon (Spearman r = 0.33, P = 3.2e-08) and rectum adenocarcinoma (Spearman r = 0.32, P = 0.0019), thymoma (Spearman r = 0.51, P = 3.4e-09), testicular Germ Cell Tumors (Spearman r = 0.53, P = 4.4e-11), and Uveal melanoma (UM) (Spearman r = 0.50, P = 2.2e-06) (**Supplementary Figure 1C**). Further Western Blot examining the expression of AhR and its downstream target gene CYP1b1 in LO2 and HCC cell lines showed that cell lines with TDO2 overexpression express relative higher level of AhR and CYP1b1 than the cell line with low expression of TDO2 (**Supplementary Figure 1D**), providing a clue that TDO2 probably regulated AhR in a translational level, which needs further work to explore.

Furthermore, addition of 50 μM exogenous Kyn resulted in a significant restore in the migration and invasion abilities of sh-Tdo2 Huh7 cells, which was counteracted by 10μM AhR antagonist CH-223191 (**Figure 5F**). Sh-Tdo2 Huh7 cells treated with exogenous Kyn showed decreased expression of E-cadherin and increased expression of N-Cadherin, MMP9 and Vimentin to different levels. These changes were reversed by AhR antagonist (**Figure 5G**). These data strongly suggested that TDO2 promoted HCC EMT through Kyn-AhR pathway.

## Discussion

HCC is one of the top life-threaten cancers worldwide, with a fearsome rate of recurrence that reaches 60–70% with 5 years and impedes the long survival of patients, despite comprehensive therapies have been applied to treat advanced HCC in clinic (3). Therefore, the fundamental mechanism of the malignant biological feature of HCC underlying the metastasis and invasion requires further exploration. TDO2 has been demonstrated to have immunomodulatory functions in promoting tumor immune resistance, which drew increasing attention to target this pathway for cancer immunotherapy (8, 30, 31). Some data have revealed that cancer cells can escape immune surveillance by overexpressing TDO2 and activating AhR in a range of cells of both the innate and adaptive immune system—dendritic cells, macrophages, natural killer cells, innate lymphoid cells, cytotoxic T cells and regulatory T cells (32, 33). Recently, TDO2 has been verified to strongly expressed in various cancers, including glioma, breast cancer, lung cancer, esophageal squamous cell carcinoma (ESCC), and could affect cancer biological features, including proliferation and metastasis, directly (9, 10, 34–36). Overexpression of TDO2 in triple negative breast cancer facilitated anoikis resistance and enhanced the metastatic capability of breast cancer cells *in vivo* (9). TDO2 was overexpressed in tumor tissue specimens obtained from UM hepatic metastasis and could be associated with the development and growth of metastasis in UM (34). TDO2 was demonstrated positively expressed in HCC (8), but there was no study for defining the role of TDO2 played in HCC. Here, we examined the effect of TDO2 on the metastasis of HCC and found that highly expression of TDO2 was related to advanced stage or invasion capabilities in cancers and enhanced migration and invasion capabilities of HCC cells both *in vitro* and vivo. The study in ESCC also corroborates with our findings in that inhibition or knockdown of TDO2 suppressed ESCC cell line proliferation and invasion (10).

EMT has been commonly considered as an important mechanism of migration and invasion for most cancer cells and related to prognosis and treatment of metastatic cancers. An altered expression of EMT markers, in particular low E-cadherin, is involved in an aggressive, malignant phenotype and early disease recurrence in HCC (11). Four EMT genes, including E-cadherin and MMP9, were found to be predictive of clinical overall survival and disease-free survival in a cohort of 128 HCC patients (37), and this was further confirmed in studies involving different centers and cohorts (38). Sorafenib, which inhibits STAT3 and phosphorylates TGF-β that are both up-regulated in EMT, is being considered as a potential therapeutic agent in HCC, but adverse events and resistance limited the therapeutic effectiveness (4). In our study, upregulated E-cadherin and downregulated N-cadherin, Vimentin and MMP9 induced by knockdown or inhibition of TDO2 were observed in HCC cells, as well as a negative correlation between TDO2 and E-cadherin and a positive correlation between TDO2 and N-cadherin in HCC samples, suggesting that TDO2 overexpression promoted HCC metastasis through inducing EMT in HCC cells. This result shed a new light on TDO2 on the development of EMT for the metastasis in HCC.

Considerable evidence supports the critical role of AhR in induction of EMT (19–22). AhR participates in the induction of Slug expression, which represses E-cadherin expression. The expression of MMPs is also a target of AhR pathway. TCDD exposure up-regulated the expression and activity of MMP9 in various malignancies including melanoma cells (39), urothelial cancer cell (40), prostate cancer cell (41), and gastric cancer cell (42). AhR was involved in the induction of EMT by Polychlorinated biphenyls in HCC cells (21). Kyn-AhR pathway has been in intensive focus recent years. Kyn has been considered to be a potent agonist of AhR, which can regulate the differentiation and activity of immune cells and thus suppress the immune response against tumors, leading to tumor immune tolerance (30). Besides, Kyn activating AhR regulated cancer growth and invasion in some malignancies (6, 22, 43). The study carried by Venkateswaran N showed that Kyn was elevated and functioned as an oncometabolite in colon cancer by promoting proliferation of colon cancer cells (43). Our *in vitro* results revealed that TDO2 was the main enzyme catalyzing Trp to Kyn in HCC cell lines, and Kyn activated AhR promoted migration and invasion capabilities through regulating EMT of HCC cell lines. Our finding is comparable with the observation published previously showing that kyn induced AhR activation enhanced invasiveness in thyroid cancer cells (22). The underlying molecular mechanism of the interaction between AhR and EMT markers and clinical relevance of this pathway remains unclear and warrants further investigation.

In conclusion, our study shows for the first time that highly expression of TDO2 is related to advanced stage and malignant traits in HCC and promotes migration and invasion capabilities of HCC cells by Kyn- AhR mediated induction of EMT. Further exploration of this pathway will provide a novel perspective into the mechanism of HCC metastasis.

## Data Availability Statement

The datasets presented in this study can be found in online repositories. The names of the repositories can be found in the article/**Supplementary Material**.

## Ethics Statement

The studies involving human participants were reviewed and approved by the ethics committee of Shanghai General hospital. The patients/participants provided their written informed consent to participate in this study. The animal study was reviewed and approved by the ethics committee of Shanghai General Hospital.

## Author Contributions

LL and TW carried out the studies, participated in collecting data, performed the statistical analysis, and drafted the manuscript. SL helped with the cell experiments. ZC and QW contributed to the animal experiments. JW and WC participated in collecting the clinical samples. XQ polished the manuscript. JX designed and supervised the study. All authors contributed to the article and approved the submitted version.

## Funding

This study was supported by funding from the National Natural Science Foundation of China (Nos. 81670595, No. 81970568).

## Conflict of Interest

The authors declare that the research was conducted in the absence of any commercial or financial relationships that could be construed as a potential conflict of interest.

## Supplementary Material

Supplementary Figure 1 **(A)** TDO2 expression in stomach adenocarcinoma and esophageal carcinoma in different stages shown by data from TCGA. **(B)** The expression level of TDO2 in gastric carcinoma in stage I–II and stage III–IV tested by Western Blot. **(C)** The correlationship of TDO2 and AhR in several cancers analyzed by data from TCGA database. **(D)** AhR and CYP1b1 expression in LO2 and 5 HCC cell lines tested by Western Blot.
