# Supplementary figures and images for: TDO2 Promotes the EMT of Hepatocellular Carcinoma Through Kyn-AhR Pathway
Source: Front Oncol. 2021 Jan 19;10:562823. doi: 10.3389/fonc.2020.562823 (PMC7851084; doi:10.3389/fonc.2020.562823)

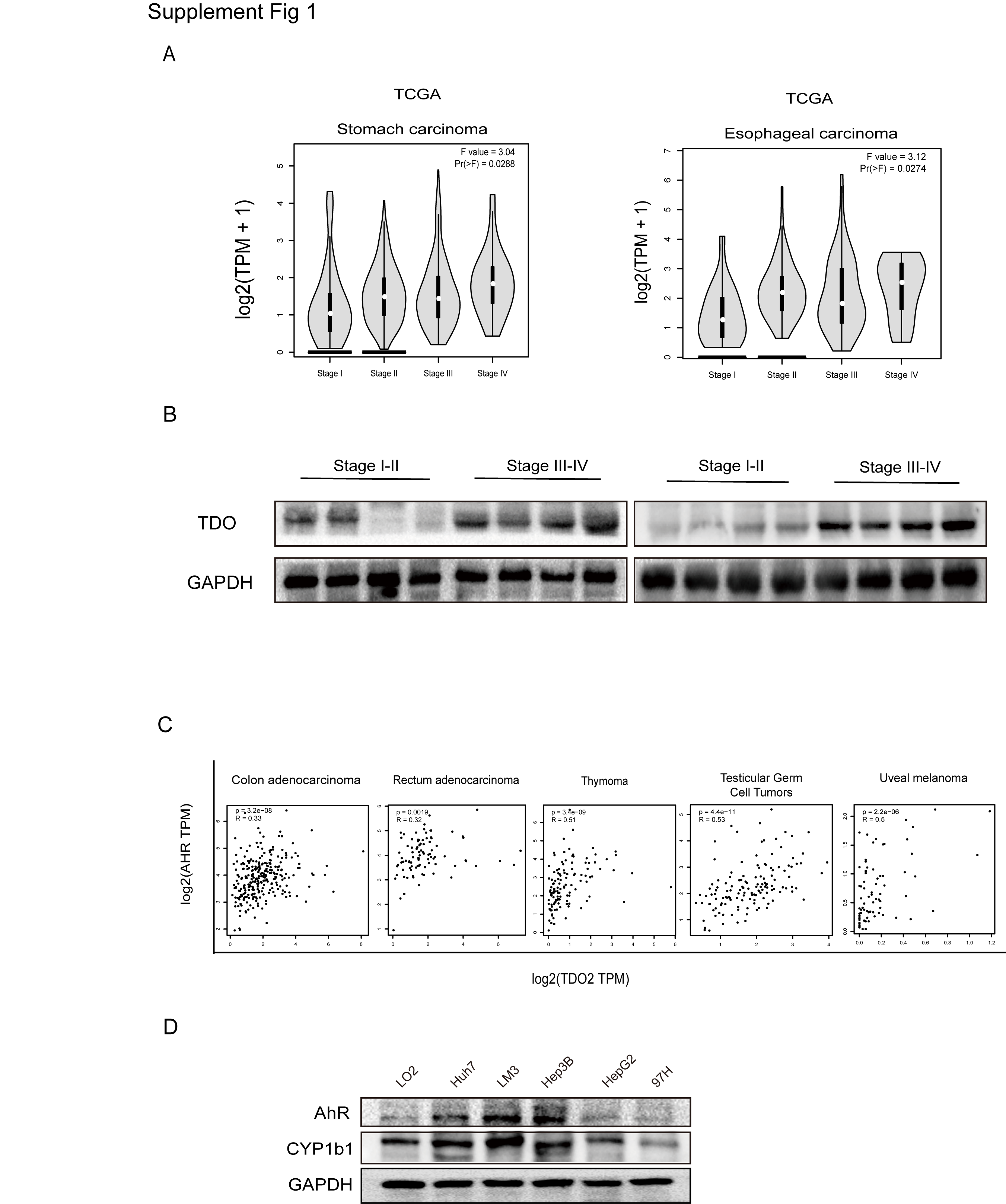

Supplement: Supplementary Figure 1 — (A) TDO2 expression in stomach adenocarcinoma and esophageal carcinoma in different stages shown by data from TCGA. (B) The expression level of TDO2 in gastric carcinoma in stage I–II and stage III–IV tested by Western Blot. (C) The correlationship of TDO2 and AhR in several cancers analyzed by data from TCGA database. (D) AhR and CYP1b1 expression in LO2 and 5 HCC cell lines tested by Western Blot. [file Image_1.tif]
