# Supplementary material for: TDO2 Promotes the EMT of Hepatocellular Carcinoma Through Kyn-AhR Pathway
Source: Front Oncol. 2021 Jan 19;10:562823. doi: 10.3389/fonc.2020.562823 (PMC7851084; doi:10.3389/fonc.2020.562823)
Supplement: Supplementary file 2 [file Table_1.docx]

Supplement Table 1. Basic information of patients

|  | N | TDO2 expression | | P value |
| --- | --- | --- | --- | --- |
|  |  | High (%) | Low (%) |  |
| Age (year) |  |  |  | >0.999 |
| <60 | 18 | 6 (33.3%) | 12 (66.7%) |  |
| >=60 | 5 | 1 (20.0%) | 4 (80.0%) |  |
| Gender |  |  |  | >0.999 |
| Male | 21 | 7 (33.3%) | 14 (66.7%) |  |
| Female | 2 | 0 (0.0%) | 2 (100.0%) |  |
| Tumor number |  |  |  | 0.0657 |
| Single | 14 | 2 (14.3%) | 12 (85.7%) |  |
| Multiple | 9 | 5 (55.6%) | 4 (44.4%) |  |
| Tumor size |  |  |  | >0.999 |
| <5 cm | 11 | 3 (27.3%) | 8 (72.7%) |  |
| >=5cm | 12 | 4 (33.3%) | 8 (66.6%) |  |
| TNM Stage |  |  |  | 0.137 |
| I - II | 16 | 3 (18.8%) | 13 (81.2%) |  |
| III -IV | 7 | 4 (57.1%) | 3 (42.9%) |  |
| Vascular invasion |  |  |  | 0.019 |
| Yes | 10 | 6 (60.0%) | 4 (40.0%) |  |
| No | 13 | 1 (7.7%) | 12 (92.3%) |  |
| Liver cirrhosis |  |  |  | 0.052 |
| Yes | 15 | 7 (46.7%) | 8 (53.3%) |  |
| No | 8 | 0 (0.0%) | 8 (100%) |  |
| Hepatitis virus |  |  |  | >0.999 |
| Yes | 20 | 6 (30.0%) | 14(70.0%) |  |
| No | 3 | 1 (33.3%) | 2 (66.7%) |  |
